# A multicenter retrospective cohort analysis of therapeutic hypothermia in acute liver failure

**DOI:** 10.1186/cc13390

**Published:** 2014-03-17

**Authors:** C Karvellas, R Stravitz, H Battenhouse, V Durkalski, W Lee, ML Schilsky

**Affiliations:** 1 University of Alberta, Edmonton, Canada; 2 Virginia Commonwealth University, Richmond, VA, USA; 3 Medical University of South Carolina, Charlston, SC, USA; 4 University of Texas Southwestern, Dallas, TX, USA; 5 Yale University, New Haven, CT, USA

## Introduction

Cerebral edema is a severe and life-threatening complication in acute liver failure (ALF). Concerns exist that therapeutic hypothermia (TH) may increase the risk of infection, worsen coagulopathy and inhibit hepatic regeneration. We therefore reviewed the experience in use of TH in participating US Acute Liver Failure Study Group (ALFSG) centers. The aims were to determine utilization of TH in ALF patients at high risk for cerebral edema (grade III or IV hepatic encephalopathy (HE)) and to determine its effect on survival and complication rates.

## Methods

A retrospective cohort study of all ALF patients enrolled in the US ALFSG registry between January 1998 and September 2013 with grade III or IV HE. TH using an external cooling device was used in 97 (8%) patients while in 1,135 (92%) patients it was not (controls).

## Results

TH ALF patients were younger (36 vs. 40, *P *= 0.03) and had acetaminophen etiology (63 vs. 47%, *P *= 0.04). Admission MELD (32 vs. 34) and lactate (5.4 vs. 5.0 mmol/l, *P *> 0.2 for all) were similar. More TH ALF patients received renal replacement therapy (63 vs. 40%), vasopressors (61 vs. 43%) and ICP monitoring (40 vs. 22%, *P *< 0.0002 for all). Overall (38% vs. 40%, *P *= 0.7) and 21-day transplant survival (45 vs. 49%, *P *= 0.5) were similar. There were no differences in bleeding (12% vs. 12%) or bloodstream infections (17 vs. 18%, *P *> 0.7 for both). More TH ALF patients had arrhythmias (38 vs. 27%, *P *= 0.03). There were no differences in listing (43 vs. 40%, *P *= 0.5) or receipt of transplant (18 vs. 25%, *P *= 0.1). After controlling for MELD, requirement for organ support on multivariable analysis, hypothermia was not independently associated with 21-day spontaneous survival (*P *= 0.93) while MELD (*P *< 0.0001) and vasopressors (*P *< 0.02) were.

## Conclusion

TH was not associated with increased bleeding/infection rates nor differences in survival in ALF patients with high-grade HE. ICP monitoring was not universally used in TH ALF patients (~40%). There is a need for a prospective trial to clarify the use of TH in patients with ALF.

